# A comparative metabolomics study of anthocyanins and taste components in Chinese bayberry (*Morella rubra*) with different flesh colors

**DOI:** 10.7717/peerj.13466

**Published:** 2022-05-31

**Authors:** Qihua Lin, Qiuzhen Zhong, Zehuang Zhang

**Affiliations:** Fruit Research Institute, Fujian Academy of Agricultural Sciences, Fuzhou, China

**Keywords:** Chinese bayberry, Primary metabolites, Anthocyanins, Flavor, Saccharides, Organic acids

## Abstract

The Chinese bayberry (*Morella rubra* Sieb. et Zucc.) is grown commercially in China and other Asian countries for its flavorful and appealing fruit. Here, two bayberry varieties differing in both color and flavor, namely, BDK (‘Baidongkui’) and DK (‘Dongkui’), in China were compared. A total of 18 anthocyanins, three proanthocyanidins, and 229 primary metabolites were identified in the pulp of the two varieties; these were analyzed and compared using ultra-performance liquid chromatography-tandem mass spectrometry. The DK pulp showed higher concentrations of all 18 anthocyanins compared with BDK, apart from peonidin-3,5-*O*-diglucoside which was not detected in BDK and which was responsible for the formation of pink pulp in BDK. Principal component analysis and cluster analysis of the primary metabolites indicated that the two bayberry varieties had distinct metabolite profiles with approximately 37% (85/229) of the primary metabolome being significantly different. Of these, 62 metabolites were down-regulated and 23 metabolites were up-regulated in BDK relative to DK. Our results suggested that the flavor of the BDK fruit was different from DK, which could be explained by the reduced saccharide, organic acid, amino acid, and proanthocyanidin contents. These findings enhance our understanding of the metabolites responsible for color and taste differences in the Chinese bayberry.

## Introduction

The Chinese bayberry (*Morella rubra* Sieb. et Zucc.) is a commercially farmed subtropical tree endemic to southern China and other regions of Asia (Chen et al., 2004; Sun et al., 2013). The fruit has many varieties, including white-, pink-, red-, purple-red-, and purple-black-fleshed fruit depending on the anthocyanin content (Jia et al., 2015; Niu et al., 2010; Sun et al., 2012a; Zhang et al., 2009). Generally, the flavor of the dark fruits is more intense than that of the light-colored fruits (Chen et al., 2016; Feng et al., 2012; Zhang et al., 2013).

Primary metabolites contribute significantly to the taste and quality of fruit. For example, saccharides and organic acids are responsible for sweet and sour tastes, respectively, while amino acids act as substrates for polyphenol oxidases associated with the browning reaction in fruit (Mertens, Mai & Glomb, 2019), as well as contributing to both flavor and fruit quality through their role as precursors of volatile compounds responsible for aroma (Gonda et al., 2010; Song & Bangerth, 2003). Flavonoid secondary metabolites contribute to both the development and quality of plants (Winkel-Shirley, 2001), with anthocyanins and proanthocyanidins forming the major flavonoid classes present in fruit (Saito et al., 2013; Sakaguchi et al., 2008). Anthocyanins contribute to fruit color, including orange, red, pink, or blue colors, while proanthocyanidins are responsible for flavor and astringency (Shi et al., 2021). Both classes represent good sources of natural antioxidants that have a role in reducing cardiovascular disease and assist with the treatment of diabetes (Bao et al., 2005; Middleton, Kandaswami & Theoharides, 2000; Shi et al., 2018a; Sun et al., 2012a; Sun et al., 2012b; Zhang et al., 2015). Despite its economic value and health benefits, the fruit of the Chinese bayberry has not been investigated in terms of metabolomics, and the metabolites responsible for color variation remain largely unknown. It is thus important to investigate the metabolomic profiles of Chinese bayberry varieties to assist the development of new and improved fruit characteristics.

The Baidongkui (BDK) variety is a new mutant line with pink fruit flesh that was identified from the Dongkui (DK) population with a similar genetic background but a different fruit color (Lin, Zhong & Zhang, 2019). The fruits color of these two varieties are the darkest in the outermost, and the color gradually decreases toward the core, and the flesh near the core is almost colorless. The fruits of these two varieties are both nearly round, with an average fruit weight of 22.0 g and a soluble solid content of 11.6%, but BDK fruit has a different flavor than DK. As it is well-known that anthocyanin synthesis depends on multiple primary metabolites (Lin et al., 2011; Niu et al., 2010; Tanaka, Sasaki & Ohmiya, 2008; Zhang et al., 2013), we were interested in the metabolite changes that affect the flavor profile of the fruit, and whether the alteration of flavor in BDK was related to the difference in color. Here, metabolomics based on ultra-performance liquid chromatography-tandem mass spectrometry (UPLC-MS/MS) was used to investigate the primary metabolites in the ripe pulp of the two Chinese bayberry varieties. Examination of the anthocyanins and proanthocyanins present was performed to determine the specific metabolites and active components influencing the taste and quality of the Chinese bayberry. It is hoped that these findings will assist in the development of novel varieties as well as contribute to the processing and consumption of the fruit.

## Materials and Methods

### Plant materials and samples

**Figure 1:**
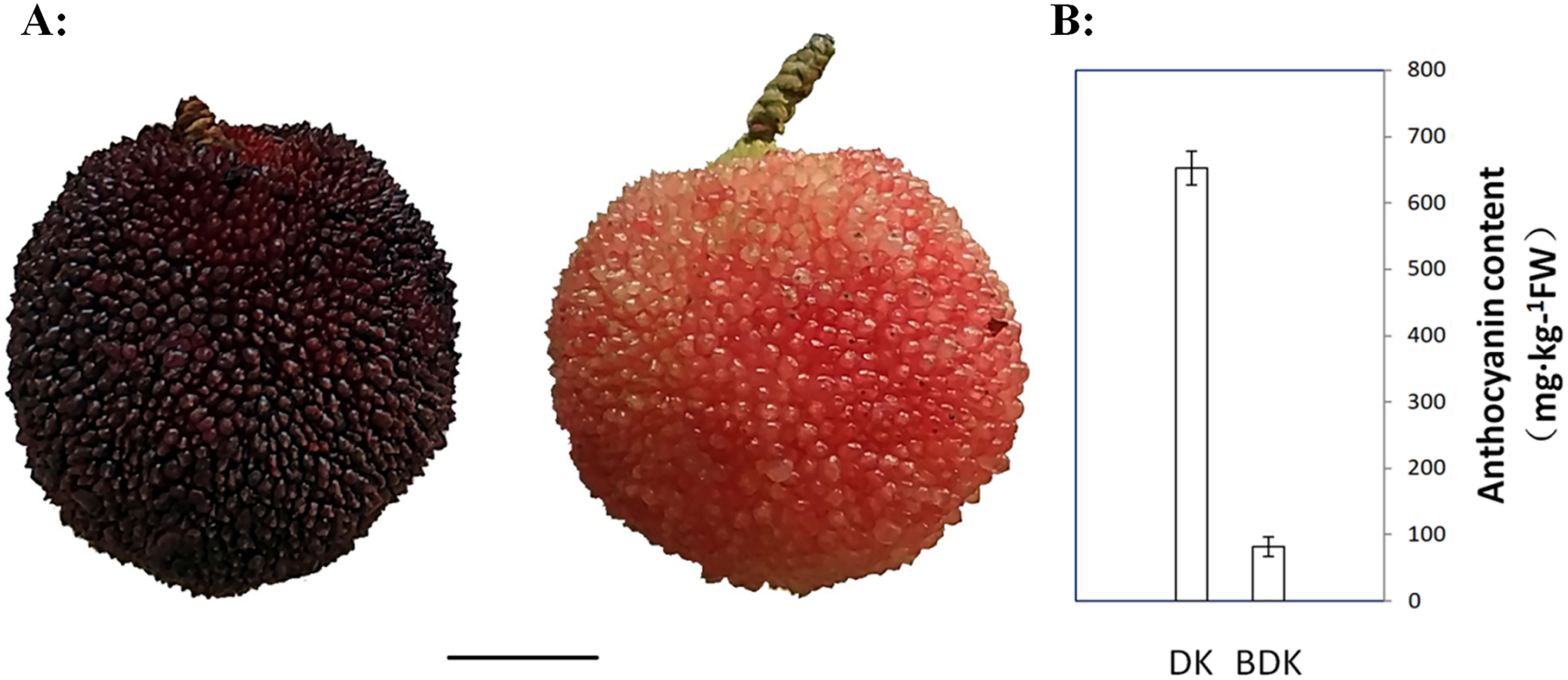
Mature Chinese bayberry fruits and anthocyanins contents of DK (purple-red-fleshed) and BDK (pink-fleshed) varieties. (A) Visual appearance of Chinese bayberry DK and BDK at ripening stage. Scale bar = 1 cm. (B) Anthocyanin content of DK and BDK fruits at ripening stage. The vertical bars represent the standard error of three biological replicates.

Two Chinese bayberry varieties, BDK and DK, were studied. BDK has pink flesh, while DK has purple-red-colored flesh (Fig. 1A). The fruit was collected from nine-year-old trees at the Fruit Research Institute, Fujian Academy of Agricultural Science, Fuzhou, China (latitude 26°07′32.96″N; longitude 119°20′15.25″E). The Chinese bayberry trees were cultivated in open fields using standard horticultural procedures, including disease and pest control.

Ten mature fruits (87 d after flowering, with the color index of red grapes values (CIRG) indices of 4.95–5.05 and 2.50–2.60 for the ripe DK and BDK fruits, respectively) were picked from each of three trees, enucleated, and the flesh amalgamated into one biological sample. Three biological samples (a total of 30 fruits) were used for each of the Chinese bayberry varieties. All samples were quickly frozen in liquid nitrogen and stored at −80 °C.

### Total anthocyanin analysis

The total anthocyanin concentration was measured by a spectrophotometric pH differential technique, as previously described (Romero et al., 2008), 1.00 g of bayberry pulp was fully grind it into powder in liquid nitrogen, added 5 mL of pre-cooled extraction solution (methanol containing 0.05% hydrochloric acid), extracted at 4 °C for 12 h, centrifuged at 10,000×*g* and 4 °C 20 min. The supernatant was collected and transferred to a clean test tube, 5 mL of extract was added to the precipitate, and leaching at 4 °C for 6 h, the supernatant was collected, and the process was repeated once. The resulting supernatants were pooled as the final measured volume. Three measurements were made for each replicate and the data were expressed as the means ± standard deviations (SD) of the three replicates.

### Anthocyanin and proanthocyanin extraction and multiple reaction monitoring (MRM)

Experimental methods and data collection as previously described (Yang et al., 2022; Yuan et al., 2018; Zhu et al., 2020). The samples were freeze-dried in a vacuum freeze-dryer. The dried samples were then pulverized in a mixer mill with a zirconia bead for 1.5 min at 30 Hz. The anthocyanins and proanthocynadins were extracted from 50 mg of powdered samples with 0.5 mL methanol/water/hydrochloric acid (500:500:1, V/V/V). The material was vortexed for 5 min and ultrasonicated for a further 5 min, after which it was centrifuged at 12,000 g for 3 min at 4 °C. This procedure was repeated once. The supernatant was retained, filtered, and subjected to UPLC-MS/MS analysis. Main parameters: solvent system, water (0.1% formic acid): methanol (0.1% formic acid); the gradient program was set to: 95:5 V/V at 0 min, 50:50 V/V at 6 min, 5:95 V/V at 12 min, hold 2 min, 95:5 V/V at 14 min; hold 2 min; temperature, flow rate and injection volume were set at 40 °C, 0.35 mL/min and 2 μL, respectively. The effluent was scanned with LIT and triple quadrupole (QQQ) alternatelyon a triple quadrupole-linear ion trap mass spectrometer (QTRAP), operating in positive ion modes. Main parameters: Ion spray voltage (IS) 5,500 V (positive ion), curtain gas (CUR) was set to 35 psi, the source temperature was set at 550 °C. A specific set of MRM transitions were monitored over each period, determined by the eluted metabolites.

In the detection of anthocyanins, except for pelargonidin-3-*O*-(6-*O*-malonyl-beta-*D*-glucoside) and peonidin-3-*O*-sophoroside (belonged to semi-quantitative substances), other substances had standard substances and belonged to targeted quantitative substances. The information of anthocyanin standard samples were listed in File S1.

### Primary metabolite extraction and MRM

The primary metabolites were extracted, and MRMs performed as previously described (Liang et al., 2022; Yang et al., 2022; Zou et al., 2020). After freeze-drying and pulverizing, 100 mg of the powdered material was added to 1.2 mL of 70% methanol solution, vortexed for 30 s every 30 min for a total of six times, and stored at 4 °C overnight. The material was then centrifuged at 12,000 g for 10 min and filtered. The extracts were then subjected to analysis on the UPLC-ESI-MS/MS system. The mobile phase used was composed of solvent A and solvent B, solvent A containing 0.1% formic acid in pure water, and solvent B containing 0.1% formic acid in acetonitrile. Sample analysis was performed using a gradient program with the column oven, flow rate and injection volume set to 40 °C, 0.35 mL/min and 4 μL, respectively. The effluent was scanned with LIT and triple quadrupole (QQQ) alternatelyon a triple quadrupole-linear ion trap mass spectrometer (QTRAP), operating in both positive and negative ion modes. Main parameters: Ion spray voltage (IS) 5,500 V (positive ion)/−4,500 V (negative ion), curtain gas (CUR) was set to 25 psi, ion source gas I (GSI) and gas II (GSII) were set to 50 and 60 psi, respectively, the source temperature was set at 550 °C. A specific set of MRM transitions were monitored over each period, determined by the eluted metabolites.

### Statistical analysis

All data were expressed as means ± standard deviation (SD) of three replicates and analyzed using SPSS version 22 (IBM Corp., Armonk, NY, USA). The metabolomic data were analyzed by principal component analysis (PCA) and heatmaps and processed using the web-based tool MetaboAnalyst (https://cloud.metware.cn/). Significantly upregulated and downregulated metabolites between the groups were determined by VIP >= 1 and absolute Log_2_FC (fold change) >= 1.

## Results

### Anthocyanins and proanthocyanins in the two Chinese bayberry varieties

Chinese bayberry fruits are usually white, pink, red, purple-red, and purple-black, dependent on the contents and types of anthocyanins (Jia et al., 2015; Zhang et al., 2009). To investigate the differences in the anthocyanin components of the different Chinese bayberry pulp colors, the purple-red variety DK and its pink-fleshed variant BDK were selected for the study. Visual observation of the two varieties showed that the DK fruit was significantly darker than BDK fruit (Fig. 1A). The total anthocyanin concentrations in the pulp were evaluated using a spectrophotometric pH differential method. This showed that the anthocyanin concentrations were higher in the DK variety (652 mg/kg of FW) in comparison with the BDK fruit (81.6 mg/kg of FW), resulting in the darker red color of DK fruit compared with the light pink coloration of the BDK fruit (Fig. 1B, File S2). This indicated that the anthocyanin content was specifically related to the variety and thus the genetic composition of the Chinese bayberry.

To fully explore the anthocyanin composition of the two Chinese bayberry varieties, the DK and BDK pulp extracts were analyzed by MRM. Eighteen anthocyanins, including cyanidin-3,5-*O*-diglucoside, cyanidin-3-*O*-(6-*O*-malonyl-beta-d-glucoside), cyanidin-3-*O*-arabinoside, cyanidin-3-*O*-glucoside, cyanidin-3-*O*-sambubioside, cyanidin-3-*O*-sophoroside, cyanidin-3-*O*-xyloside, delphinidin-3-*O*-glucoside, delphinidin-3-*O*-rhamnoside, malvidin-3-*O*-arabinoside, malvidin-3-o-galactoside, malvidin-3-*O*-glucoside, pelargonidin-3-*O*-(6-*O*-malonyl-beta-d-glucoside), pelargonidin-3-*O*-glucoside, peonidin-3,5-*O*-diglucoside, peonidin-3-*O*-glucoside, peonidin-3-*O*-sophoroside, and petunidin-3-*O*-glucoside were isolated and characterized; peonidin-3,5-*O*-diglucoside was not detected in BDK. Of these 18 anthocyanins, 13 have not been identified in the Chinese bayberry fruit previously. The major anthocyanin identified was cyanidin-3-*O*-glucoside (Table 1), in agreement with previous observations (Feng et al., 2012). In addition, although the same kinds of anthocyanins were identified in both bayberry varieties, the levels of the compounds differed markedly, with the concentrations of the 18 anthocyanins in DK (with dark-colored pulp) higher than those in BDK (with lighter-colored pulp) (Table 1), suggesting the possible inhibition of genes related to anthocyanin biosynthesis or regulation in BDK, resulting in lower anthocyanin levels in the fruit. These observations were confirmed by the quantitative determination of the anthocyanin concentrations between the two varieties (Fig. 1B).

**Table 1:** Anthocyanin types and concentrations in the pulp of the two Chinese bayberry varieties.

Compounds	Molecular weight (Da)	BDK (μg.g^−1^)	DK (μg.g^−1^)	*P*
Cyanidin-3,5-*O*-diglucoside	611.16	0.0129 ± 0.000157**	0.116 ± 0.00113**	0.000
Cyanidin-3-*O*-(6-*O*-malonyl-beta-D-glucoside)	535.11	0.0255 ± 0.000456**	0.0806 ± 0.00141**	0.000
Cyanidin-3-*O*-arabinoside	419.10	0.0108 ± 0.000433**	0.0748 ± 0.00325**	0.000
Cyanidin-3-*O*-glucoside	449.11	132 ± 2.15**	824 ± 9.31**	0.000
Cyanidin-3-*O*-sambubioside	581.16	0.00566 ± 0.000488**	0.0476 ± 0.00457**	0.000
Cyanidin-3-*O*-sophoroside	611.16	0.305 ± 0.0133**	0.929 ± 0.0456**	0.000
Cyanidin-3-*O*-xyloside	419.10	0.0986 ± 0.00391**	3.45 ± 0.136**	0.000
Delphinidin-3-*O*-glucoside	465.10	4.97 ± 0.145**	26.0 ± 0.655**	0.000
Delphinidin-3-*O*-rhamnoside	419.10	0.120 ± 0.00270**	0.159 ± 0.00447**	0.000
Malvidin-3-*O*-arabinoside	463.12	0.0121 ± 0.000869**	0.0171 ± 0.00139**	0.006
Malvidin-3-*O*-galactoside	493.13	0.0117 ± 0.00108**	0.0331 ± 0.00299**	0.000
Malvidin-3-*O*-glucoside	493.13	0.0202 ± 0.000319**	0.0409 ± 0.00101**	0.000
Pelargonidin-3-*O*-(6-*O*-malonyl-beta-D-glucoside)	519.11	0.0255 ± 0.000311**	0.673 ± 0.00189**	0.000
Pelargonidin-3-*O*-glucoside	433.11	3.85 ± 0.152**	6.59 ± 0.216**	0.000
Peonidin-3,5-*O*-diglucoside	625.17	ND	0.0216 ± 0.00248	
Peonidin-3-*O*-glucoside	463.12	2.87 ± 0.0895**	23.2 ± 0.868**	0.000
Peonidin-3-*O*-sophoroside	625.18	0.0697 ± 0.000397**	0.730 ± 0.00379**	0.000
Petunidin-3-*O*-glucoside	479.12	0.214 ± 0.00788**	0.522 ± 0.0171**	0.000

**Notes:**

The unit of anthocyanin content is μg.g^−1^, values are means ± standard deviations of means from three repeats.

** In each row indicate significant difference between two varieties (*P* < 0.01).

Three proanthocyanins were identified and analyzed in the pulp of the two Chinese bayberry varieties by MRM. These included procyanidin A2, procyanidin B2, and procyanidin C1. DK was rich in both procyanidin A2 and procyanidin C1, with the concentration of procyanidin A2 in DK being 5.11 times that of BDK, while BDK had a comparable procyanidin B2 content (Table 2).

**Table 2:** Proanthocyanidin types and concentrations in the pulp of the two Chinese bayberry varieties.

Compounds	Molecular weight (Da)	BDK (μg.g^−1^)	DK (μg.g^−1^)	*P*
Procyanidin A2	576.1268	0.0354 ± 0.00304**	0.181 ± 0.0203**	0.000
Procyanidin B2	578.1424	6.89 ± 0.703	6.65 ± 0.625	0.686
Procyanidin C1	866.2058	1.52 ± 0.106*	2.20 ± 0.304*	0.022

**Notes:**

The unit of proanthocyanidin content is μg.g^−1^, values are means ± standard deviations of means from three repeats.

* In each row indicate significant difference between samples (*P* < 0.05).

** In each row indicate significant difference between two varieties (*P* < 0.01).

### Primary metabolites in the two Chinese bayberry varieties

To better understand flavor and quality differences between the two varieties, we performed widely targeted UPLC-MS/MS-based metabolite profiling of the differently flavored DK and BDK. A total of 229 metabolites were identified, many of which were likely to have contributed to taste (14 saccharides and alcohols, 41 organic acids, and 47 amino acids and derivatives), together with other primary metabolites (Files S3 and S4).

**Figure 2:**
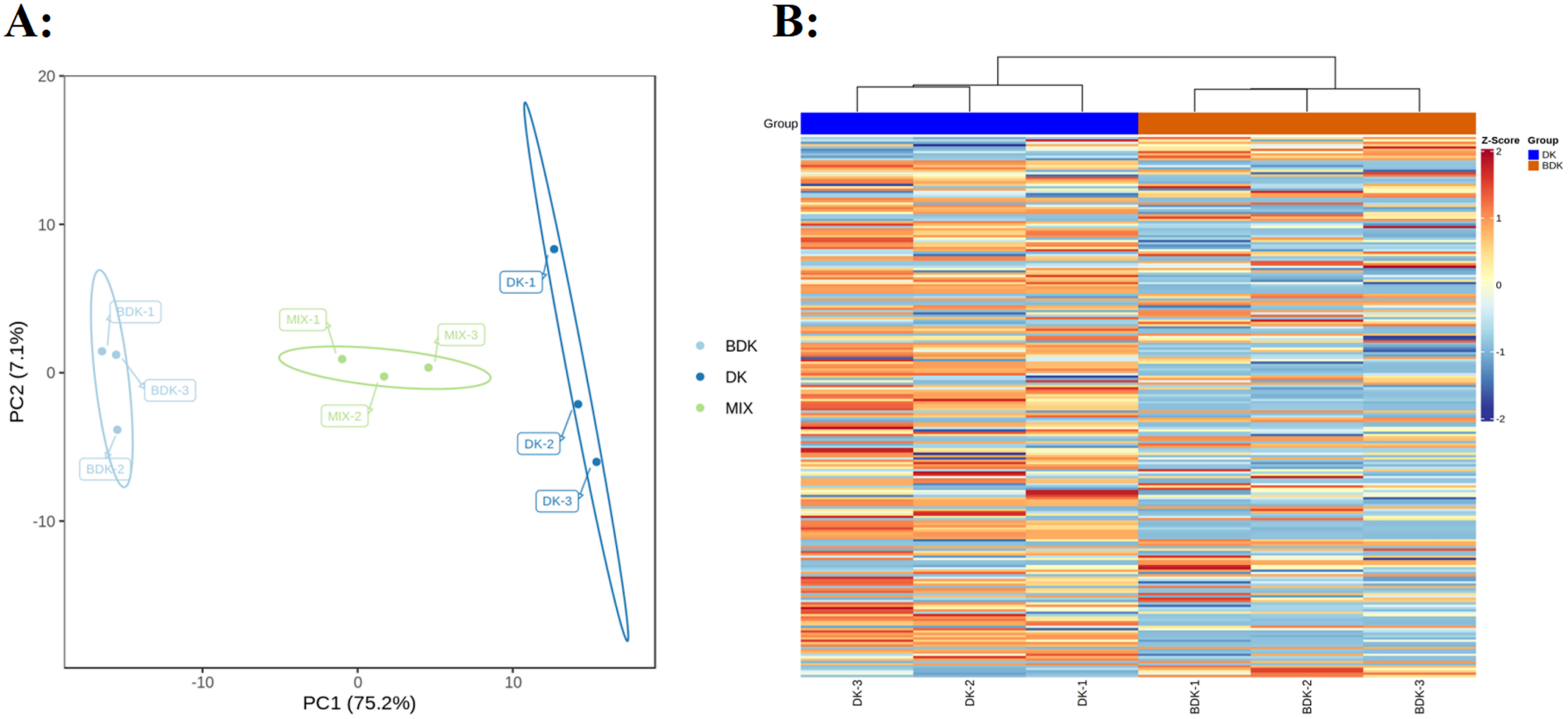
Overview of metabolic profiles of DK and BDK. (A) PCA analysis of metabolites identified of DK and BDK. Equal volumes of the two varieties fruit samples were mixed used for quality control (MIX). (B) Cluster analysis of metabolites of the two varieties. The color indicates the level of accumulation of each metabolite, from low (blue) to high (brown).

PCA of the 229 metabolites showed a clear separation between the DK and BDK samples (Fig. 2A), with the first PC accounting for over 75% of the total variability. We used a log10 transformation of the metabolite peaks, followed by hierarchical cluster analysis, to remove the influence of quantity on pattern recognition. As shown by the heatmap (Fig. 2B), the biological replicates from each variety were clustered, indicating the reliability of the metabolic profiles. Thus, both PCA and cluster analysis demonstrated distinct metabolic profiles for the two varieties.

### Differentially accumulated primary metabolites in the fruit of the two varieties

**Figure 3:**
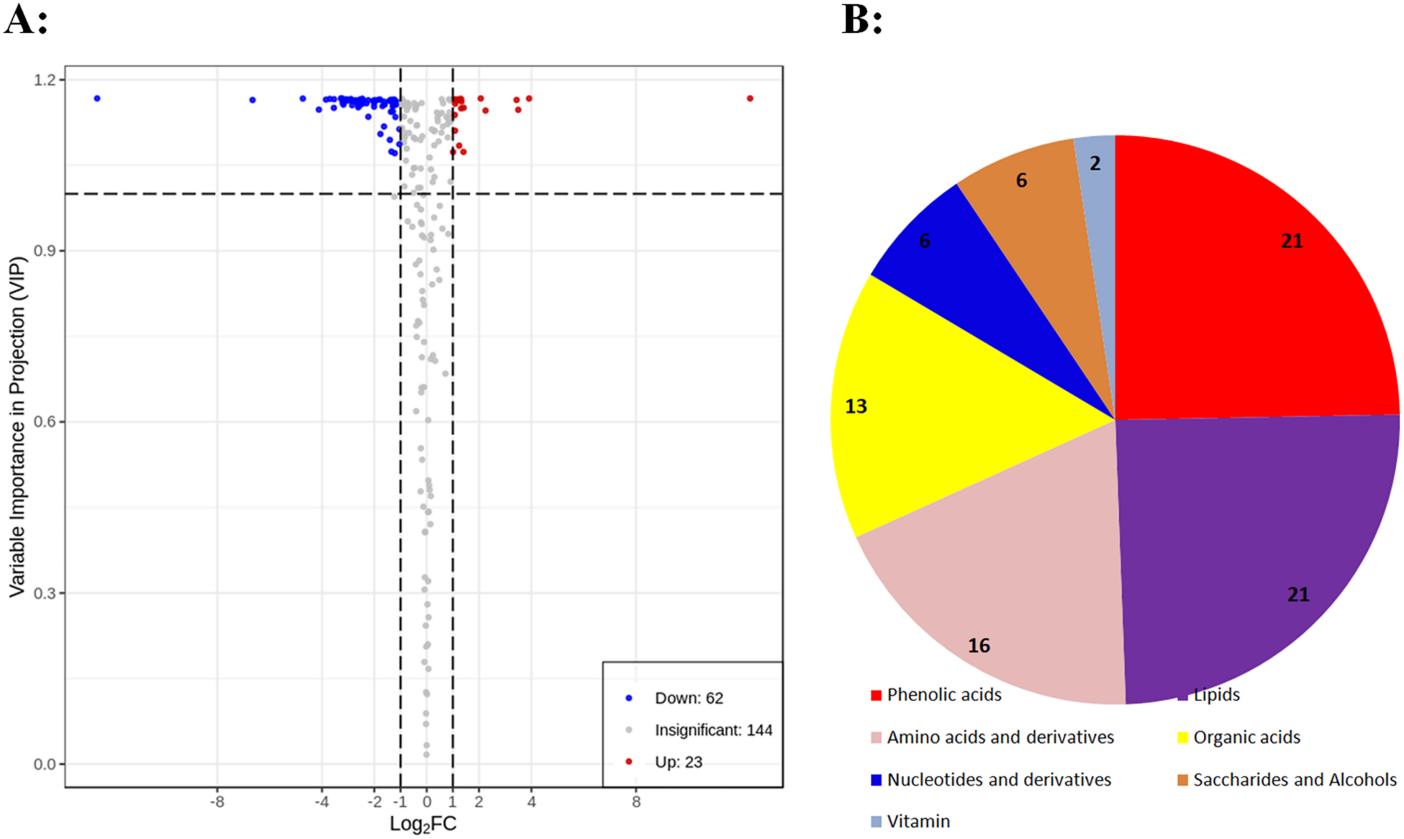
Differentially accumulating primary metabolites between DK and BDK. (A) Volcano plot of the 229 metabolites identified. Differential metabolites were defined as metabolites with fold change ≥ 2.0 or ≤ 0.5 in BDK compared to DK. A threshold of VIP ≥ 1.0 was used to separate differential metabolites from unchanged metabolites. (B) Pie chart depicting the biochemical categories of the differential primary metabolites identified between DK and BDK.

To identify differential primary metabolites between DK and BDK, metabolites with fold changes ≥ 2.0 (upregulated) or ≤ 0.5 (downregulated) were selected. The metabolites were analyzed using a variable importance in projection (VIP) value (VIP ≥ 1.0), resulting in the identification of 85 differential metabolites between the varieties (Fig. 3A). In all, 37% of the total 229 metabolites were found to be differentially expressed, indicating significant differences in the metabolite contents of the two varieties. Of these, 62 metabolites were downregulated in BDK compared with DK, while 23 were upregulated (Fig. 3A). These 85 metabolites belonged to seven classes, with most being phenolic acids, lipids, amino acids and amino acid derivatives, and organic acids (Fig. 3B).

### Associations of saccharides, organic acids, and other components with fruit taste and quality

The Chinese bayberry fruit pulp contained primarily fructose, glucose, and sucrose. In all, 17 saccharides were detected in the two varieties. To the best of our knowledge, this is the first identification of these saccharides in the pulp of the Chinese bayberry. It was found that the overall sweet taste of the fruit was dependent not only on the concentrations of the various saccharides but also on their relative proportions. The DK variety had relatively higher levels of fructose and glucose and a lower level of sucrose compared to BDK (File S3), while the concentrations of D-glucoronic acid, gluconic acid, D-sorbitol, and mannitol in DK were over twice those seen in BDK, with D-fructose 6-phosphate and D-glucose 6-phosphate levels of less than one-half those of BDK (Table 3).

**Table 3:** Statistics of differentially accumulating saccharides, organic acids and amino acids in the pulp of DK and BDK varieties.

Class	Compounds	Peak area		Fold change	VIP (≥1.0)	*P*	Type
		DK	BDK				
Saccharides	Gluconic acid	17,424,667 ± 632,412**	655,853 ± 36,657**	0.04	1.17	0.000	down
	D-Glucoronic acid	1,397,600 ± 126095**	150,597 ± 31,672**	0.11	1.16	0.000	down
	D-Sorbitol	216,393 ± 10,930**	88,387 ± 12,956**	0.41	1.14	0.000	down
	Mannitol	44,223 ± 3,865**	5,256 ± 751**	0.12	1.16	0.000	down
	D-Glucose 6-phosphate	466030 ± 9908**	1,063,000 ± 48,756**	2.28	1.16	0.000	up
	D-Fructose 6-phosphate	183,463 ± 21,559**	488,980 ± 37,427**	2.67	1.15	0.000	up
Organic acids	Anchoic acid	32,724,333 ± 1,929,107**	5,611,233 ± 363,034**	0.17	1.17	0.000	down
	4-Acetamidobutyric acid	2,432,067 ± 247,474**	606,017 ± 40,909**	0.25	1.16	0.000	down
	Muconic acid	2,267,833 ± 159,616**	732,763 ± 230,596**	0.32	1.12	0.000	down
	2-Hydroxyhexadecanoic acid	1,851,900 ± 131,491**	128,703 ± 16,261**	0.07	1.17	0.000	down
	2-Isopropylmalate	1,791,367 ± 148,565**	17,802 ± 5,249**	0.01	1.16	0.000	down
	(S)-(−)-2-Hydroxyisocaproic acid	1,145,300 ± 35,606**	320,717 ± 22,014**	0.28	1.16	0.000	down
	D-Galacturonic acid	1,070,773 ± 66,888**	167,700 ± 28,308**	0.16	1.16	0.000	down
	2-Hydroxy-4-methylpentanoic acid	64,089 ± 4,470**	31,077 ± 6,016**	0.48	1.11	0.002	down
	3-Dehydro-L-Threonic acid	3319700 ± 122,310*	7,033,500 ± 1,428,506*	2.12	1.11	0.011	up
	2,4,6-Trihydroxybenzoic acid	1,857,133 ± 161,642**	4,620,400 ± 502,837**	2.49	1.15	0.000	up
	2-Hydroxycinnamic acid	515,923 ± 13,632**	1,283,533 ± 18,239**	2.49	1.17	0.000	up
	Fumaric acid	23,545 ± 6,070**	55,668 ± 8,673**	2.36	1.08	0.006	up
	Diethyl phosphate	ND	47,315 ± 3,252				up
Amino acids and derivatives	3-Hydroxy-3-methylpentane-1,5-dioic acid	3,834,967 ± 109,080**	295,143 ± 26,234**	0.08	1.17	0.000	down
	L-(+)-Arginine	28,84,633 ± 465,023**	1,117,433 ± 58,285**	0.39	1.14	0.000	down
	L-Valyl-L-Leucine	1,057,367 ± 201,018**	116,750 ± 23,794**	0.11	1.16	0.000	down
	L-Leucyl-L-phenylalanine	1,052,403 ± 92,795**	142,287 ± 18,363**	0.14	1.16	0.000	down
	L-Leucyl-L-Leucine	625,140 ± 69,923**	113,870 ± 18,479**	0.18	1.16	0.000	down
	L-Valyl-L-Phenylalanine	448,863 ± 56,373**	72,242 ± 5,067**	0.16	1.16	0.000	down
	L-Isoleucyl-L-Aspartate	184,717 ± 34,112**	25,699 ± 4,536**	0.14	1.16	0.000	down
	L-Glycyl-L-phenylalanine	175,113 ± 20,150**	43,484 ± 6,269**	0.25	1.15	0.000	down
	L-Prolyl-L-Leucine	141,220 ± 26,854**	23,555 ± 3,668**	0.17	1.15	0.000	down
	DL-Alanyl-DL-phenylalanine	110,272 ± 25,016**	17,984 ± 2,763**	0.16	1.15	0.000	down
	N-Acetyl-L-glutamic acid	71,975 ± 11,917**	27,144 ± 6,832**	0.38	1.09	0.005	down
	N-Acetyl-L-Arginine	55,532 ± 4,165	ND				down
	L-Phenylalanyl-L-phenylalanine	50,940 ± 8,824**	8,788 ± 564**	0.17	1.16	0.000	down
	S-Adenosylmethionine	83,978 ± 10,040*	168,953 ± 37,895*	2.01	1.07	0.020	up
	Glutathione reduced form	45,473 ± 11,876**	514,260 ± 132,426**	11.31	1.15	0.000	up
	Oxiglutatione	26,320 ± 5,799*	69,723 ± 18,442*	2.65	1.07	0.018	up

**Notes:**

The relative amount of primary metabolite is calculated by calculating the peak area formed by the characteristic ions of each species in the detector. Values are means ± standard deviations of means from three repeats.

* In each row indicate significant difference between two varieties (*P* < 0.05).

** In each row indicate significant difference between two varieties (*P* < 0.01). Differentially accumulating compounds are identified using thresholds of variable importance in projection (VIP) ≥1.0 and fold change ≥ 2.0 (up-regulated) or ≤ 0.5 (down-regulated) in BDK compared to DK.

A total of 41 organic acids were detected in the two varieties, including major organic acids such as citric acid, malic acid, succinic acid, and quinic acid; of these, 37 have not been previously reported (File S3). In terms of differential metabolites, eight organic acids, namely anchoic acid, 2-isopropylmalate acid, muconic acid, D-galacturonic acid, 2-hydroxyhexadecanoic acid, 4-acetamidobutyric acid, (S)-(−)-2-hydroxyisocaproic acid, and 2-hydroxy-4-methylpentanoic acid, were found to be more abundant in DK. Fumaric acid, 3-dehydro-L-threonic acid, diethyl phosphate, 2-hydroxycinnamic acid, and 2,4,6-trihydroxybenzoic acid were found to be more abundant in BDK (Table 3).

Forty-seven amino acids and their derivatives were identified in the two varieties. Of these, 16 amino acids were differentially accumulated using thresholds of VIP ≥1.0 and fold change ≥ 2.0 (upregulated) or ≤ 0.5 (downregulated). Thirteen of these, 3-hydroxy-3-methylpentane-1,5-dioic acid, L-(+)-arginine, N-acetyl-L-glutamic acid, N-acetyl-L-arginine, L-glycyl-L-phenylalanine, L-prolyl-L-leucine, L-valyl-L-leucine, DL-alanyl-DL-phenylalanine, L-leucyl-L-leucine, L-isoleucyl-L-aspartate, L-alanyl-L-phenylalanine, L-leucyl-L-phenylalanine, and L-phenylalanyl-L-phenylalanine showed greater accumulation in DK compared with BDK (Table 3).

Phenolic acids, lipids, nucleotides and their derivatives, and vitamins were also identified. Of these, seven phenolic acids, 18 lipids, four nucleotides and derivatives, and two vitamins were present in greater concentrations in DK than BDK, as determined by thresholds of VIP ≥1.0 and fold change ≥ 2.0 (File S5).

## Discussion

The Chinese bayberry is an economically important fruit found in China and surrounding Asian countries. The fruit of the Chinese bayberry varies considerably in terms of color, and the fruit color is related not only to appearance but also to the nutritional value and quality (Bao et al., 2005; Shi et al., 2018b). It has been reported that the anthocyanin derivatives cyanidin and pelargonidin are responsible for the bright red coloration of the fruit, while derivatives of malvidin, petunidin, and delphinidin are associated with purple and other dark colors (Jaakola, 2013; Zhang et al., 2020). In our study, the anthocyanin components of the fruit of the purple-red bayberry variety DK and the pink bayberry variety BDK were essentially the same, with only peonidin-3,5-*O*-diglucoside not detected in BDK. However, the contents of these anthocyanin components, as well as the total anthocyanin value in BDK fruits, were significantly lower than those in DK, resulting in the pink color of the mature BDK fruit and the purple-red color of fruit from the DK variety. Our previous research confirmed inhibition of the upstream genes of the anthocyanin metabolism pathway in the BDK fruit, resulting in a decrease in the contents of total anthocyanin (Lin, Zhong & Zhang, 2019). This suggested possible inhibition of the upstream factors of the anthocyanin metabolism pathway in the BDK fruit, resulting in a decrease in the contents of each of the anthocyanin components.

Widely targeted metabolite profiling using MS/MS data has been shown to be successful in the large-scale metabolite profiling and comparative metabolomics in numerous plant species (Chen et al., 2013; Oikawa et al., 2015; Wang et al., 2016; Wang et al., 2017; Wang et al., 2018; Zou et al., 2020). Previous studies on the metabolites in the Chinese bayberry have largely focused on specific metabolites, including saccharides (fructose, glucose, and sucrose), organic acids, and amino acids in the fruit (Chen et al., 2016; Feng et al., 2012). However, there has been no comparative analysis of the comprehensive metabolic profiles of Chinese bayberry varieties. Here, we used UPLC-MS/MS-based widely targeted metabolomics to investigate variations in taste between two varieties with similar genetic backgrounds but differing in both fruit color and flavor. In all, 229 primary metabolites were identified, of which 85 were differentially accumulated between DK and BDK. Therefore, this study provided new insight into the metabolic reasons for the taste differences between pink- and purple-red-fleshed Chinese bayberry.

Generally, the flavor of fruit can be classified as sweet or sour (Crisosto et al., 2004; Minas et al., 2013; Wang, Chen & Wang, 2009). These attributes are closely related to the fruit constituents, especially saccharides and organic acids. We observed a variety of saccharides in the Chinese bayberry fruit, of which D-(+)-sucrose, D-(+)-glucose, and D-fructose were the most common, consistent with previous reports (Feng et al., 2012). Notably, we found that four of the six significantly different saccharides in BDK were present in significantly lower concentrations in this variety compared with DK. Moreover, our results indicated the most common organic acids in the Chinese bayberry, namely, L-(−)-malic acid, citric acid, and isocitric acid, were present in comparable amounts in both varieties. However, BDK contained significantly lower concentrations of 12 metabolites. Although the types of saccharides and organic acids were similar in the two varieties, the concentrations differed, with lower contents of most saccharides and organic acids in BDK relative to DK, which may explain the different taste of the BDK fruit.

Amino acids not only contribute significantly to the nutritional content but also affect the flavor of fruit (Mertens, Mai & Glomb, 2019; Phillips et al., 2015; Shiraishi, Fujishima & Chijiwa, 2009; Yoo et al., 2013). Eighteen amino acids have been previously identified in the Chinese bayberry fruit (Fang et al., 2006). However, the present study identified 47 amino acids by widely targeted metabolomics, 16 of which were differentially accumulated between DK and BDK (Table 3). Thirteen of these were significantly down-regulated and differentially accumulated in BDK (Table 1). Proanthocyanidins usually have astringent tastes (Shi et al., 2021), and differences in the types and contents of these compounds will thus affect the fruit flavor. We found that the contents of procyanidin A2 and procyanidin C1 in BDK were lower than in DK. Thus, our results suggest that the down regulation of most of the differentially accumulated amino acids and proanthocyanidins in the BDK fruit influences its flavor.

**Figure 4:**
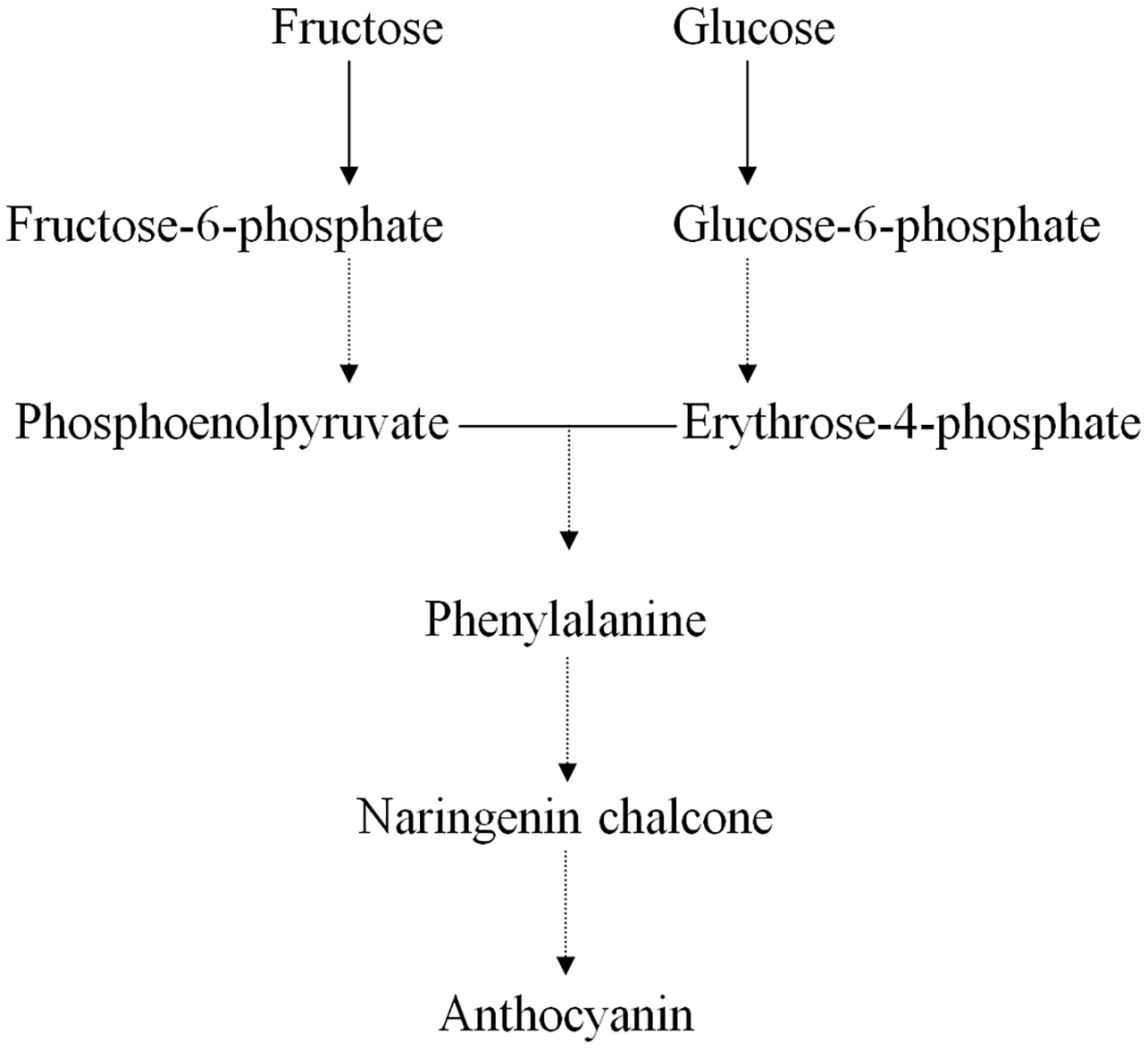
Relative pathway of anthocyanin related biosynthesis in Chinese bayberry fruit.

Earlier studies have shown that the ternary MYB–bHLH–WD40 (MBW) transcription factor complex influences the expression of genes regulating anthocyanin structure and biosynthesis in the Chinese bayberry (Liu et al., 2013a; Liu et al., 2013b). Many primary metabolites act as precursors in the synthesis of anthocyanins, such as D-glucose, D-fructose, D-fructose 6-phosphate, and D-glucose 6-phosphate (Fig. 4) (Chen et al., 2016; Feng et al., 2012; Shi et al., 2014; Zhang et al., 2013). We found that the concentrations of both D-fructose 6-phosphate and D-glucose 6-phosphate were significantly greater in BDK than in DK, while the anthocyanin content in BDK was significantly reduced compared with the DK variety. Our previous study observed a reduction in the expression of genes related to anthocyanin synthesis in BDK, leading to lower anthocyanin levels (Lin, Zhong & Zhang, 2019). We tentatively speculated that the greater accumulation of D-fructose 6-phosphate and D-glucose 6-phosphate in BDK might be due to inhibition of anthocyanin metabolism, resulting in the build up of high levels of these saccharides that cannot be further transformed. It has been observed that D-glucose 6-phosphate was present in higher levels in light-colored loquat flesh compared with darker varieties (Zou et al., 2020), although the underlying mechanism requires further investigation.

## Conclusions

In this study, a total of 18 anthocyanins, three proanthocyanidins, and 229 primary metabolites were identified and compared in the pulp of two Chinese bayberry varieties with highly similar genetic backgrounds using UPLC-MS/MS. This work also comprehensively analyzed the composition and concentrations of primary metabolites, anthocyanins, and proanthocyanidins in Chinese bayberry fruit. The results tentatively put forward that the downregulated expression of all anthocyanins present in BDK was the underlying cause of the pink-colored pulp compared with the dark-colored pulp of DK, and that differences in the composition and concentrations of saccharides, organic acids, amino acids, and proanthocyanidins may be the main cause of the differences in taste between the two varieties.

## Supplemental Information

10.7717/peerj.13466/supp-1 Supplemental Information 1 Information of anthocyanin standard samples.

10.7717/peerj.13466/supp-2 Supplemental Information 2 Raw Data: Anthocyanin content of DK and BDK.

10.7717/peerj.13466/supp-3 Supplemental Information 3 Information and contents of primary metabolites in the pulp of BDK and DK.

10.7717/peerj.13466/supp-4 Supplemental Information 4 TIC of MRM.

10.7717/peerj.13466/supp-5 Supplemental Information 5 Differentially accumulating primary metabolites in the pulp of BDK and DK.

## Additional Information and Declarations

### Data Availability

The following information was supplied regarding data availability: The raw measurements are available in the Supplementary Files.
