# Supplementary material for: A comparative metabolomics study of anthocyanins and taste components in Chinese bayberry (Morella rubra) with different flesh colors
Source: PeerJ. 2022 May 31;10:e13466. doi: 10.7717/peerj.13466 (PMC9165596; doi:10.7717/peerj.13466)
Supplement: Supplemental Information 1 [file peerj-10-13466-s001.docx]

**Information of anthocyanin standard samples**

| **CAS** | **Compounds** | **Brand** | **Remark** |
| --- | --- | --- | --- |
| 20905-74-2 | Cyanidin-3,5-*O*-diglucoside | ExtraSynthese |  |
| 171828-62-9 | Cyanidin-3-*O*-(6-*O*-malonyl-beta-D-glucoside) | Zzstandard |  |
| 27214-72-8 | Cyanidin-3-*O*-arabinoside | ExtraSynthese |  |
| 47705-70-4 | Cyanidin-3-*O*-glucoside | ANPEL |  |
| 63535-17-1 | Cyanidin-3-*O*-sambubioside | ExtraSynthese |  |
| 38820-68-7 | Cyanidin-3-*O*-sophoroside | ExtraSynthese |  |
| 29761-24-8 | Cyanidin-3-*O*-xyloside | Zzstandard |  |
| 50986-17-9 | Delphinidin-3-*O*-glucoside | Zzstandard | In the chloride form |
| 29907-19-5 | Delphinidin-3-*O*-rhamnoside | ExtraSynthese | In the chloride form |
| 785047-89-4 | Malvidin-3-*O*-arabinoside | Zzstandard |  |
| 30113-37-2 | Malvidin-3-*O*-galactoside | Zzstandard | In the chloride form |
| 18470-06-9 | Malvidin-3-*O*-glucoside | Sigma |  |
| 47684-27-5 | Pelargonidin-3-*O*-glucoside | ExtraSynthese |  |
| 47851-83-2 | Peonidin-3,5-*O*-diglucoside | ExtraSynthese | In the chloride form |
| 68795-37-9 | Peonidin-3-*O*-glucoside |  | No brand information |
| 6988-81-4 | Petunidin-3-*O*-glucoside |  | No brand information |
