# Supplementary figures and images for: A comparative metabolomics study of anthocyanins and taste components in Chinese bayberry (Morella rubra) with different flesh colors
Source: PeerJ. 2022 May 31;10:e13466. doi: 10.7717/peerj.13466 (PMC9165596; doi:10.7717/peerj.13466)

**TIC of MRM**


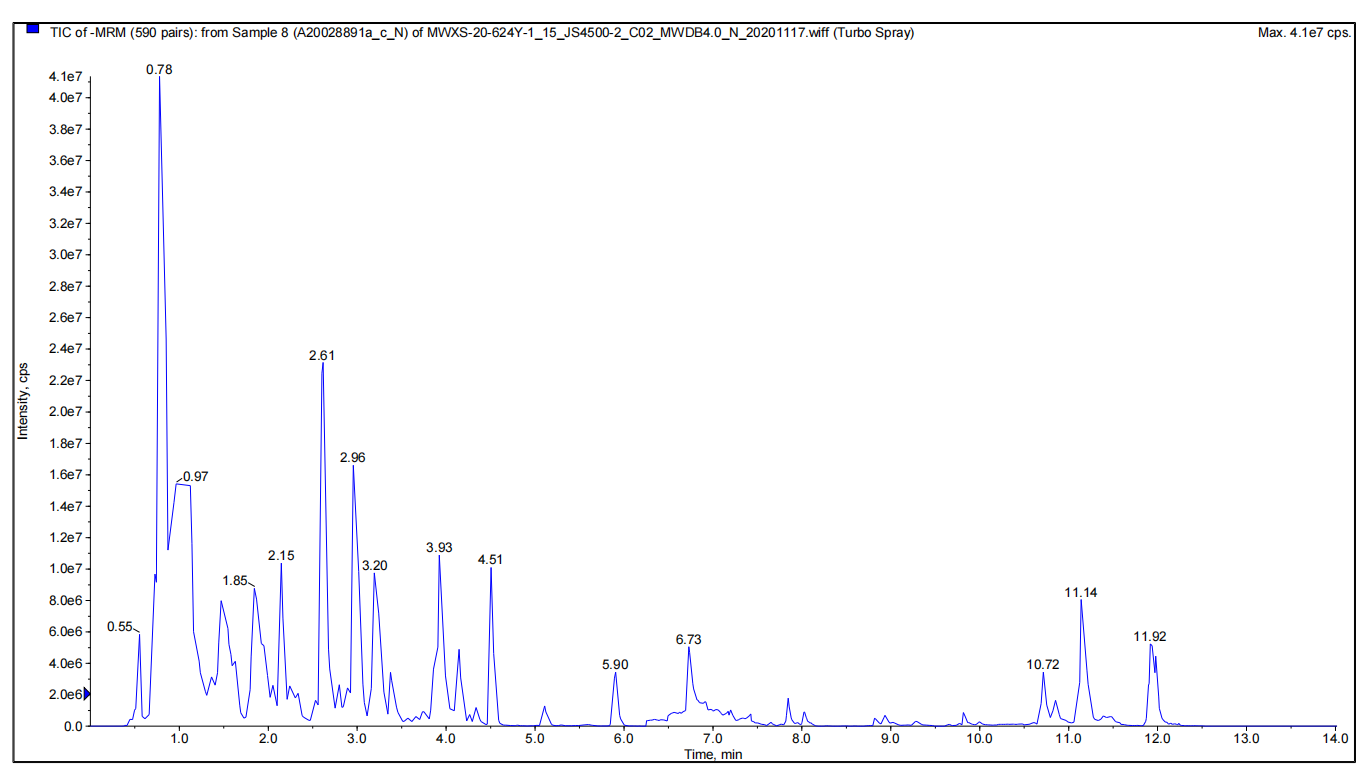

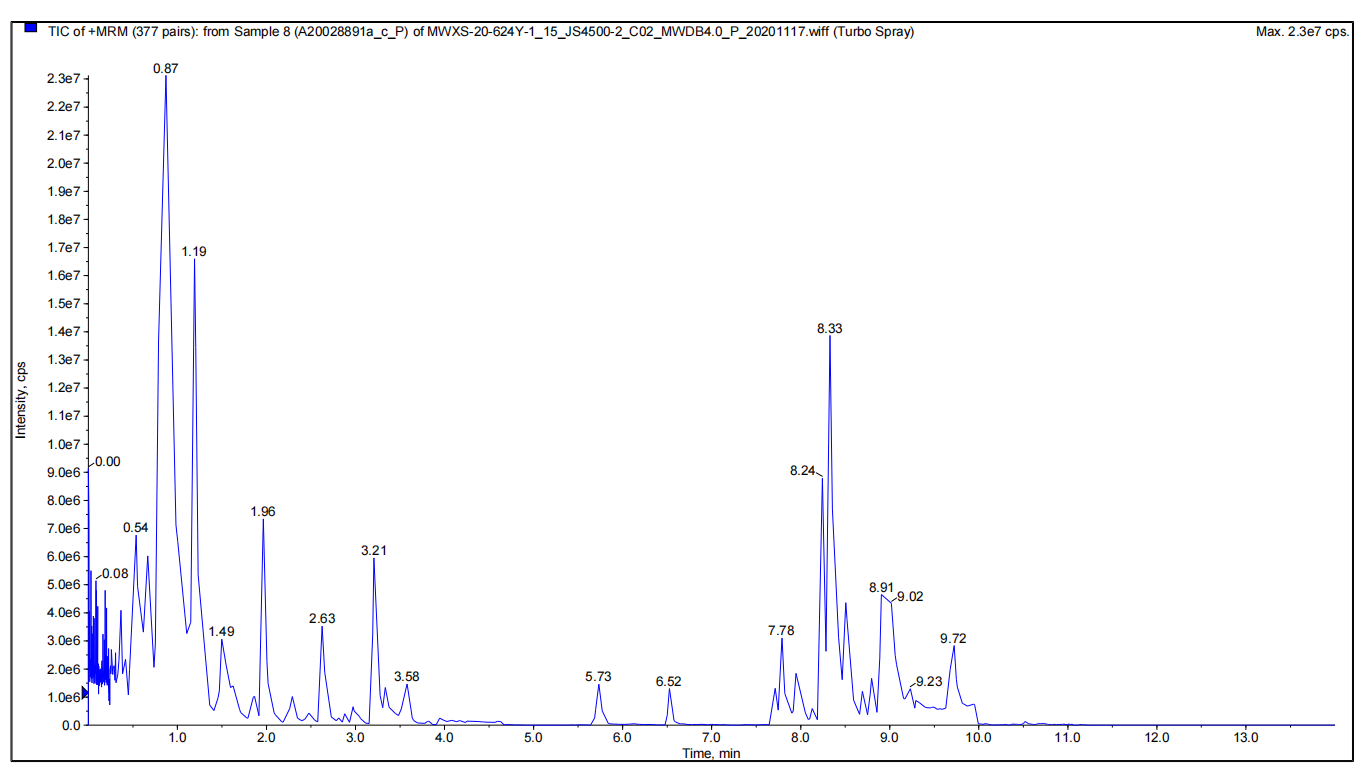


**DK-1_N DK-1_P**


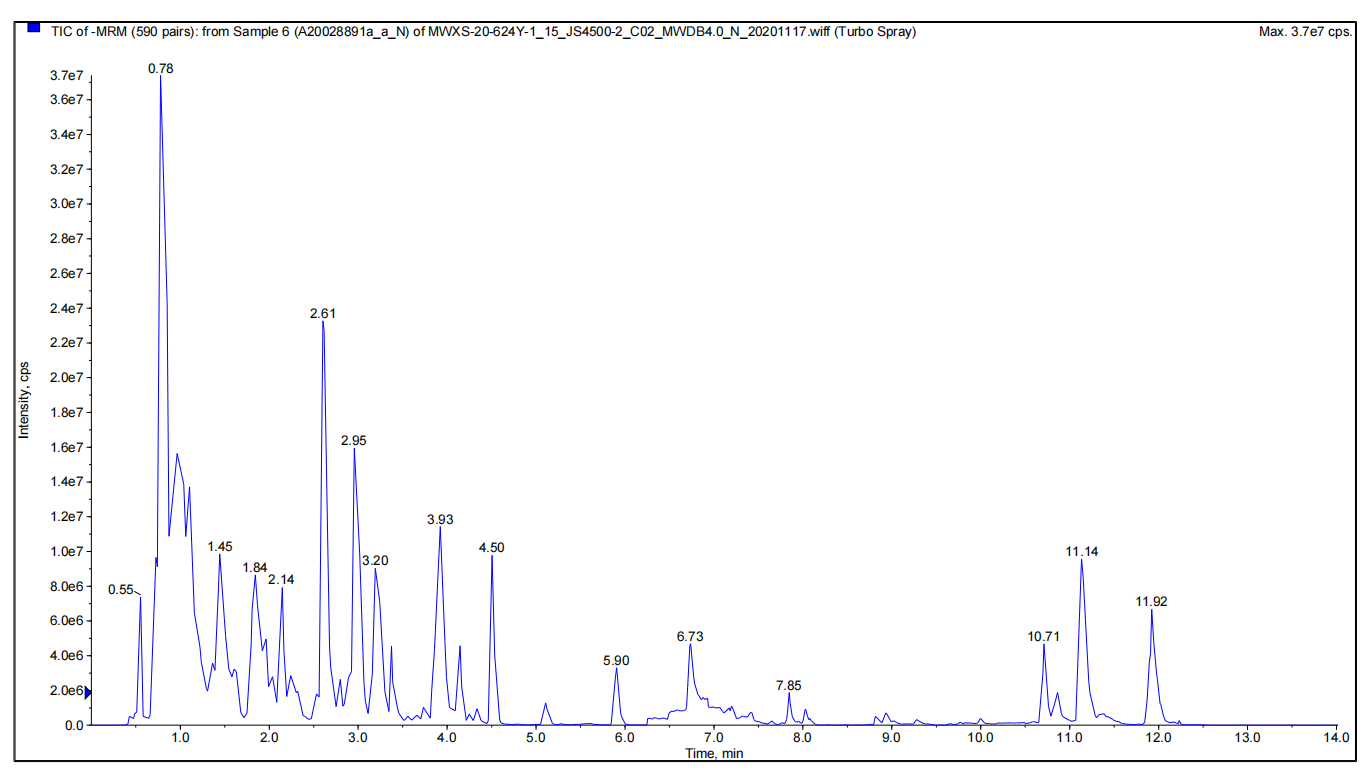

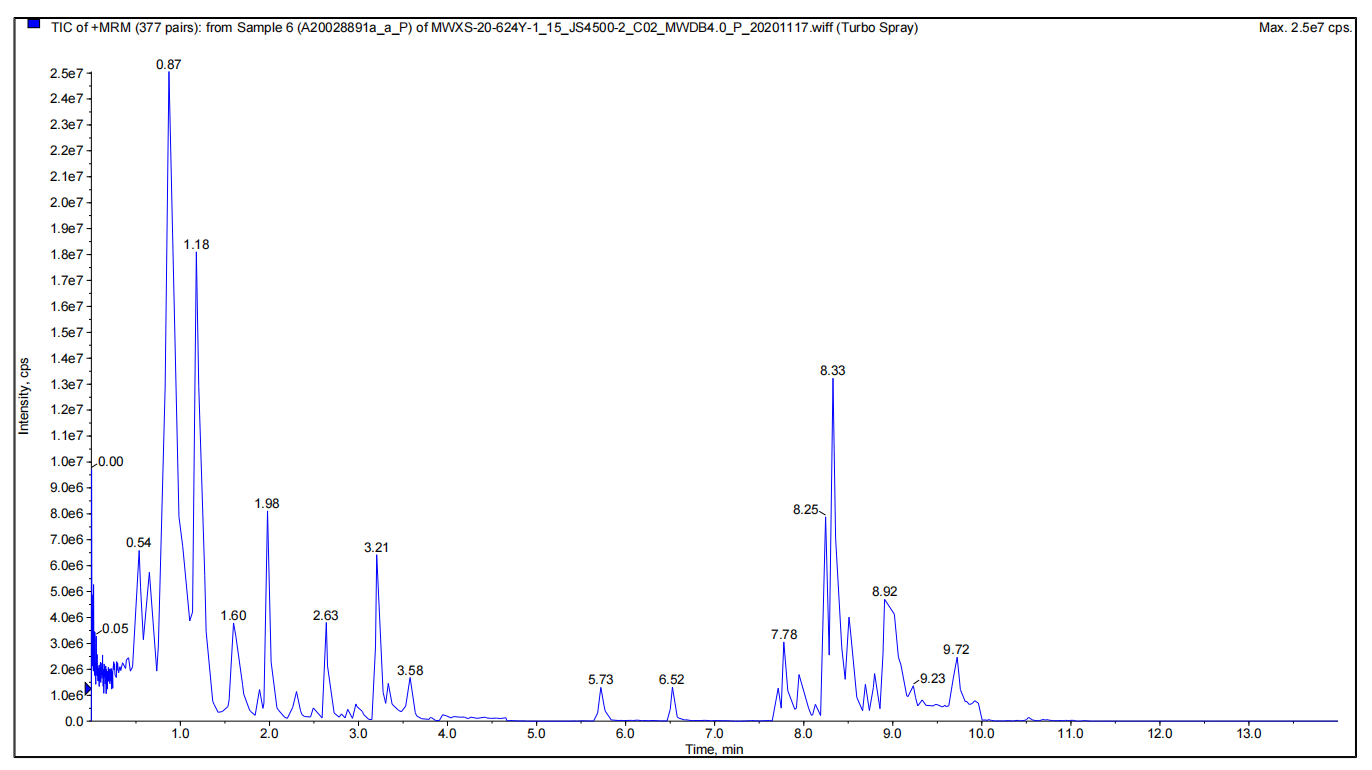


**DK-2_N DK-2_P**


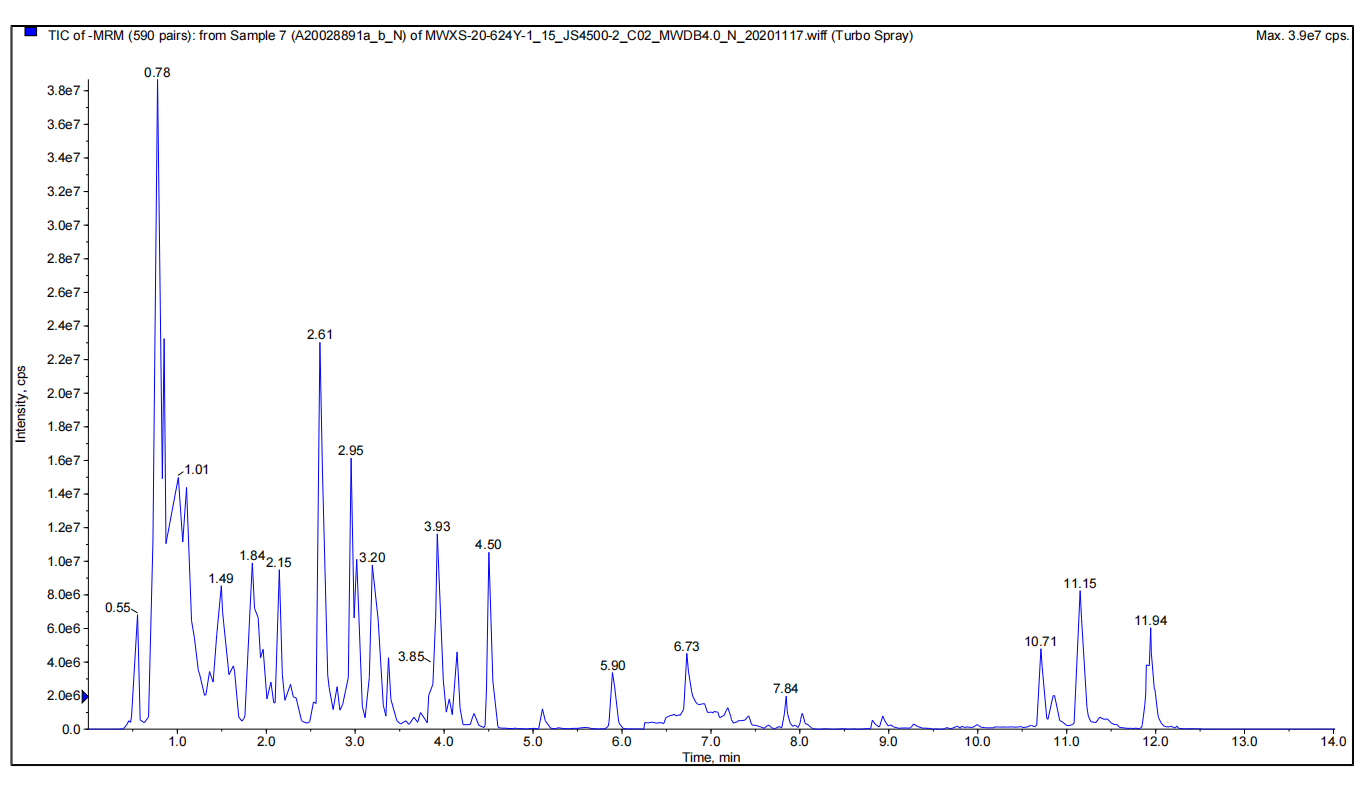

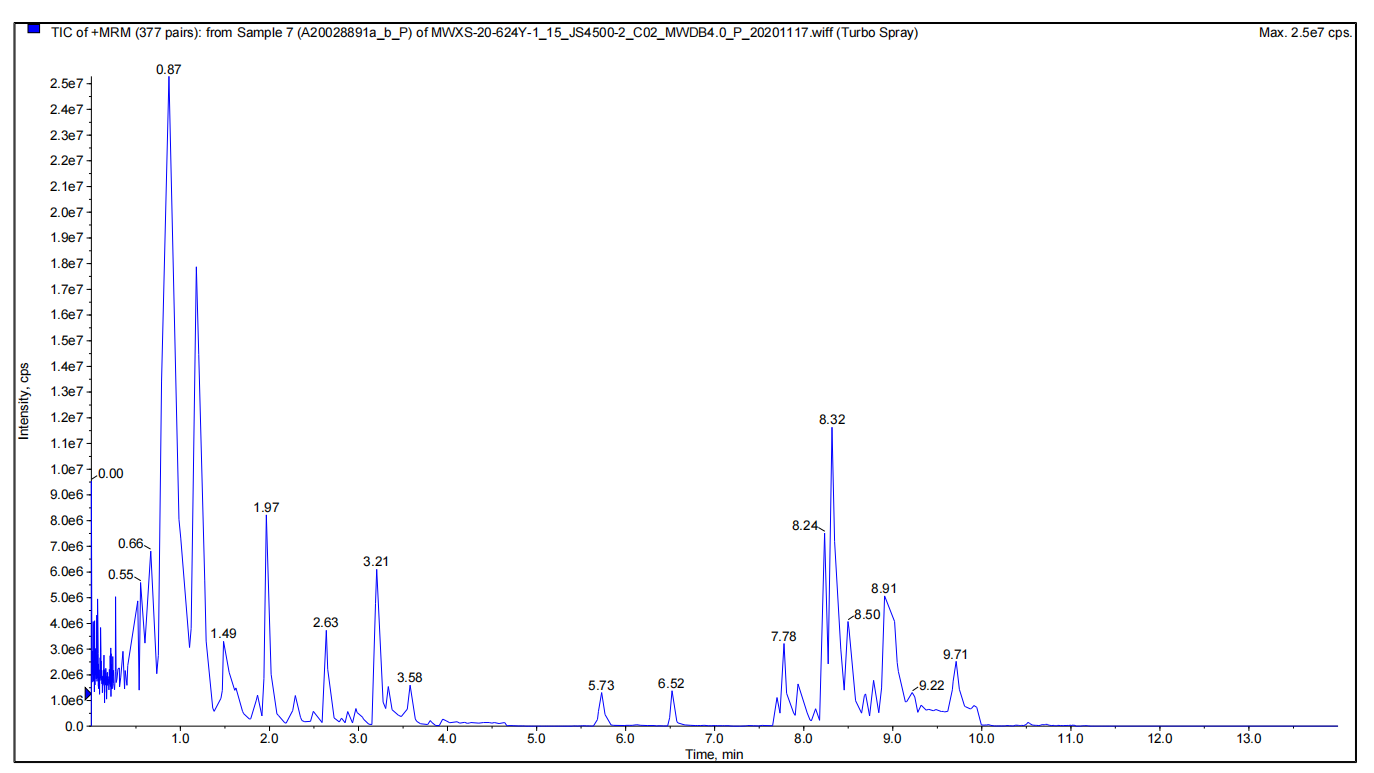


**DK-3_N DK-3_P**


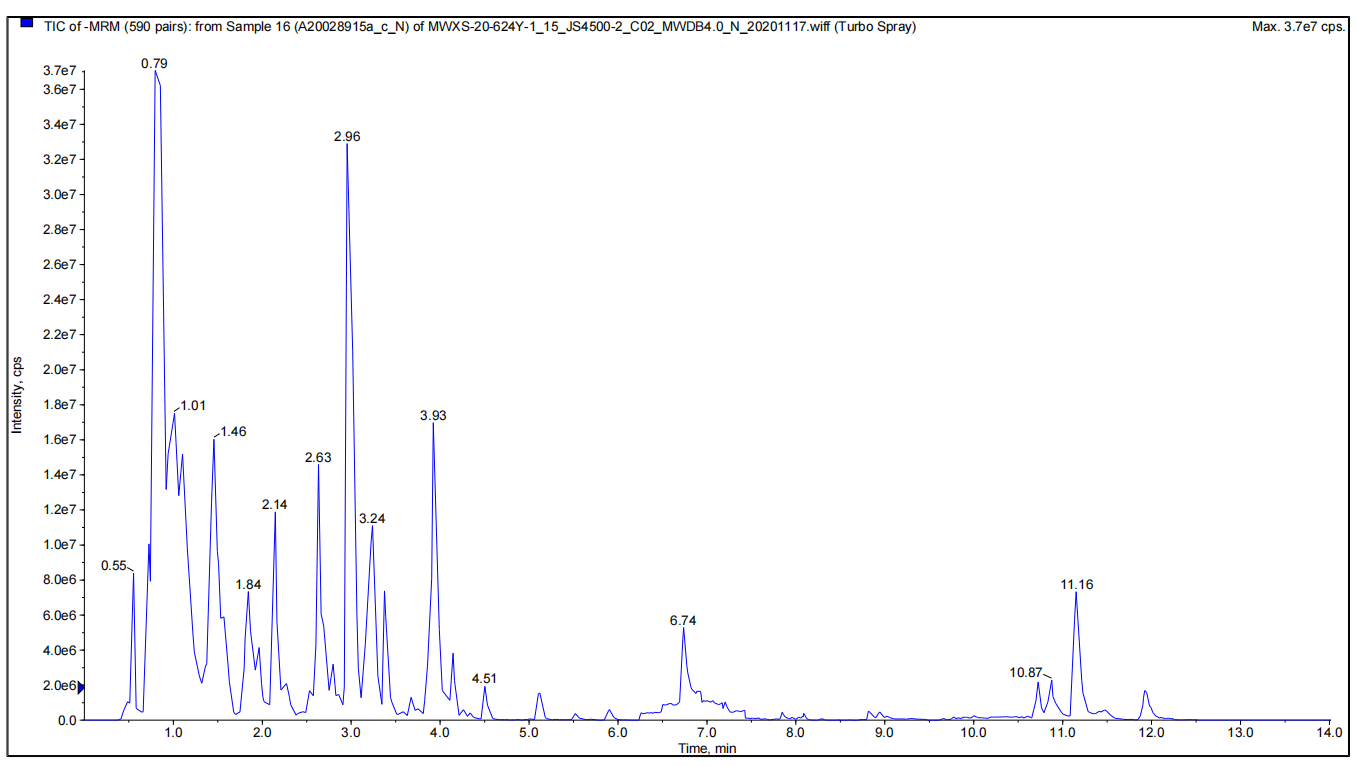

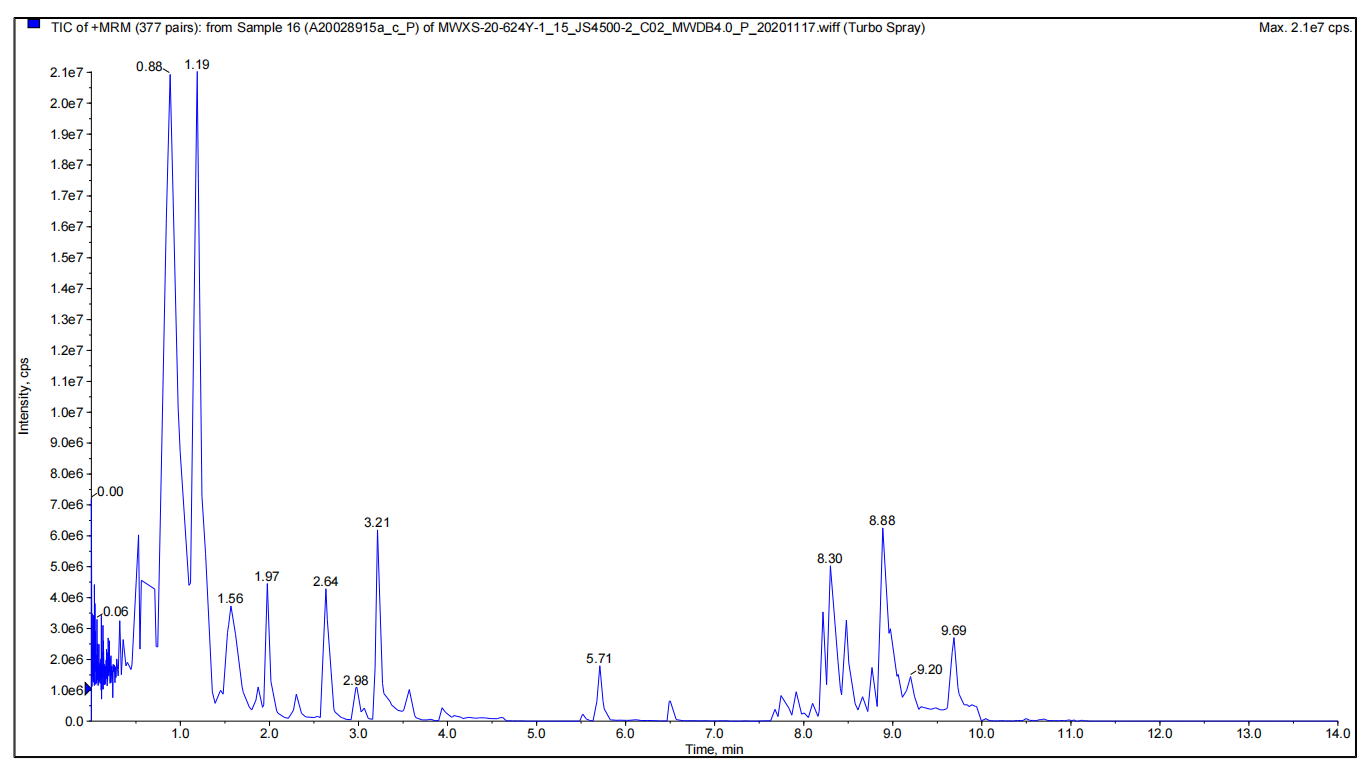


**BDK-1_N BDK-1_P**


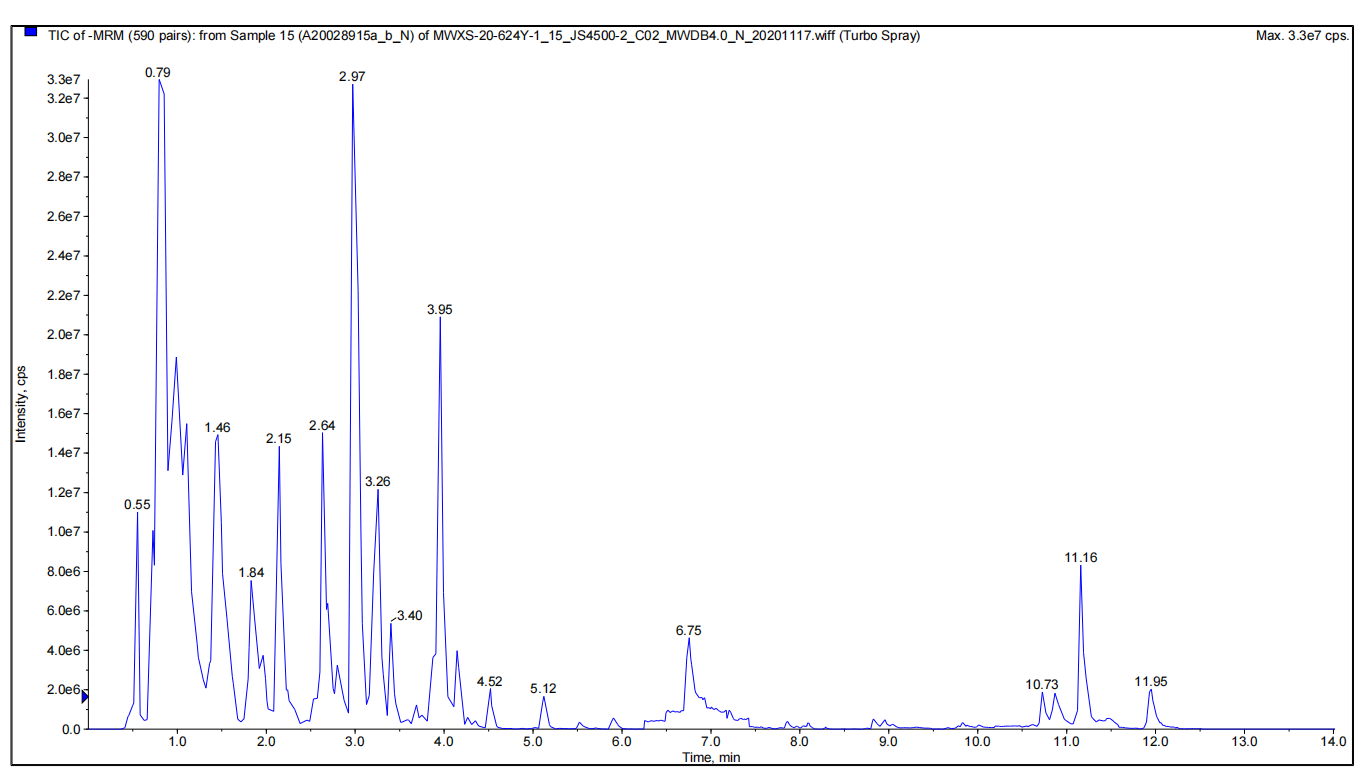

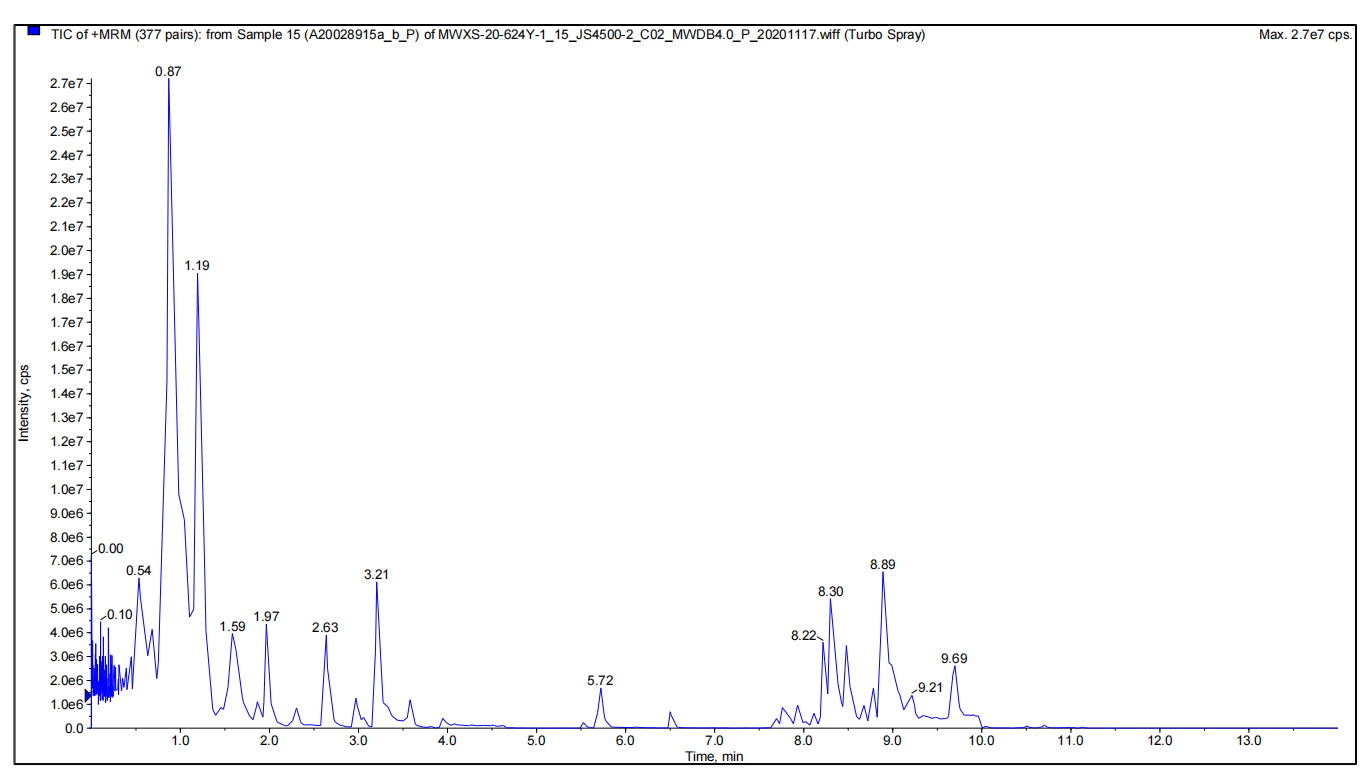


**BDK-2_N BDK-2_P**


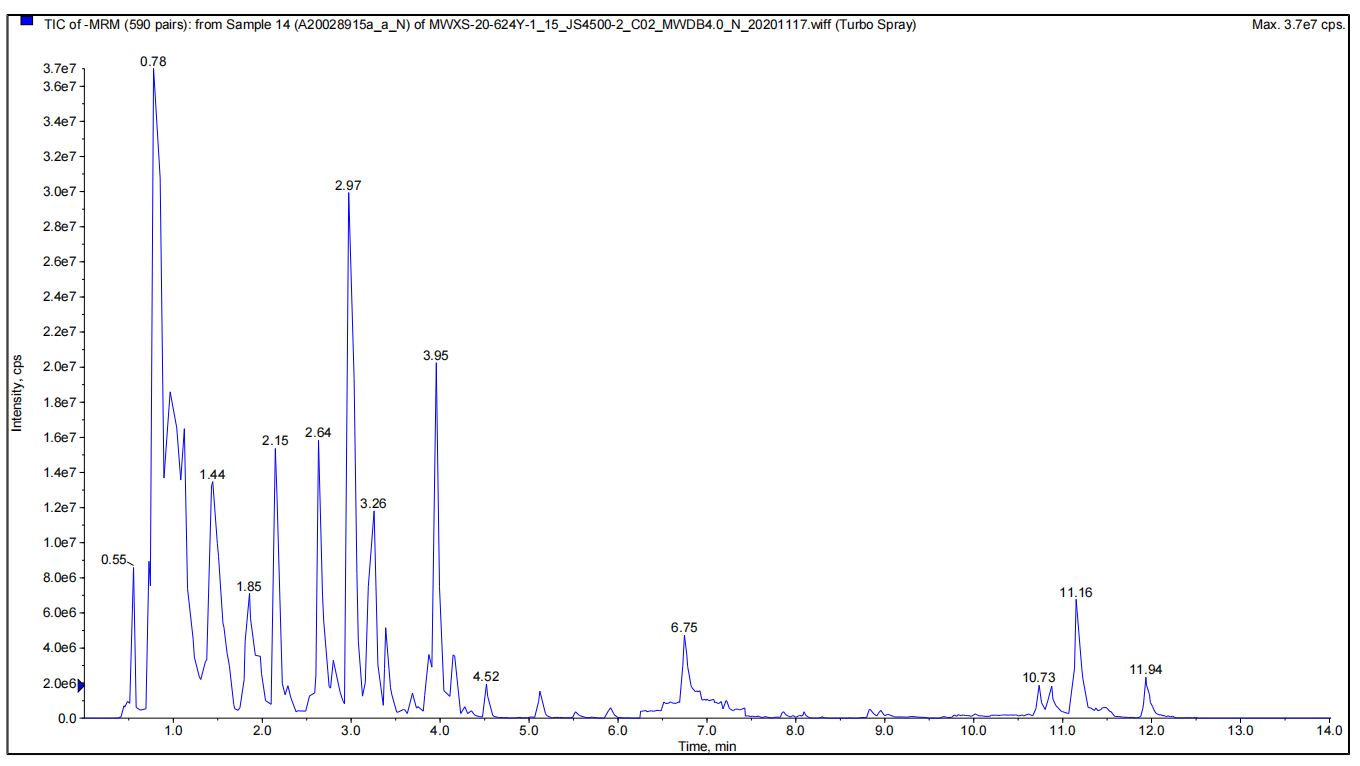

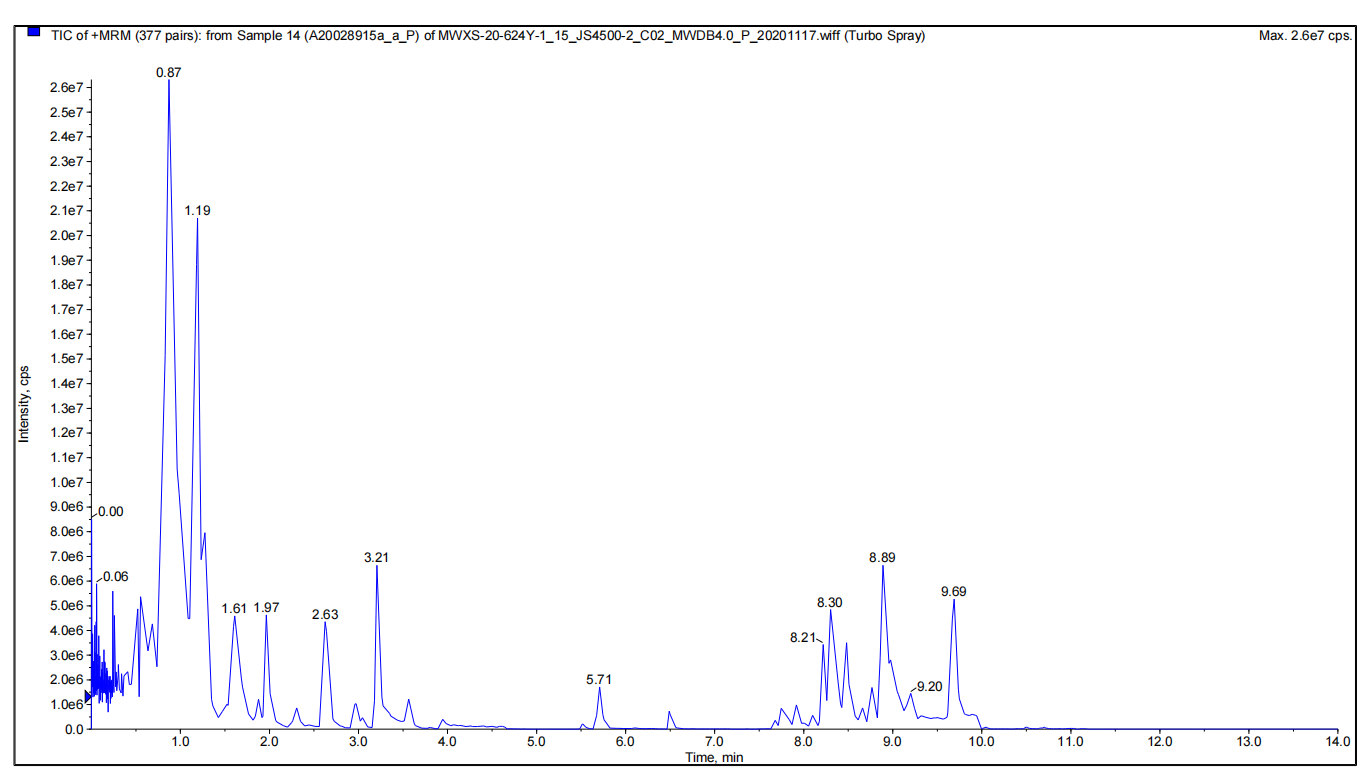


**BDK-3_N BDK-3_P**

Supplement: Supplemental Information 4 [file peerj-10-13466-s004.doc]
